# Emicizumab as first-line therapy in acquired hemophilia A

**DOI:** 10.1016/j.rpth.2024.102438

**Published:** 2024-05-13

**Authors:** Michael Iarossi, Cedric Hermans

**Affiliations:** Division of Hematology, Hemostasis and Thrombosis Unit, Saint-Luc University Hospital, Université catholique de Louvain, Brussels, Belgium

**Keywords:** acquired hemophilia A, bleeding, comorbidities, emicizumab, immunosuppressive therapy

## Abstract

Acquired hemophilia A (AHA) is a rare autoimmune disease resulting from the development of autoantibodies directed against endogenous factor (F)VIII, leading to bleeding manifestations that can be life-threatening. The current standard hemostatic treatment involves the use of bypassing agents that circumvent FVIII (recombinant activated FVII, activated prothrombin complex concentrate, and recombinant porcine FVIII) that must be administered intravenously and possess a short half-life. These limitations and the risk of potentially fatal bleeding complications justify the early initiation of immunosuppressive treatment (IST) aimed at promptly eradicating the autoantibodies. IST is not without side effects, sometimes severe and possibly fatal, especially in persons with AHA who are generally older and have multiple comorbidities. Emicizumab, a bispecific antibody that mimics the action of FVIII, has emerged as an effective hemostatic therapy among persons with congenital hemophilia, whether complicated by the presence of anti-FVIII antibodies or not. Numerous arguments from recent clinical experiences suggest positioning emicizumab as a first-line treatment for AHA. This strategy has the potential to reduce bleeding complications and, importantly, the side effects associated with IST, which can be delayed and tailored to each patient.

Acquired hemophilia A (AHA) results from the development of neutralizing autoantibodies against endogenous factor (F)VIII. Typically, it predominantly affects older individuals with comorbidities who do not have a prior history of bleeding. It is usually idiopathic but can complicate numerous pathologies. The most prevalent bleeding manifestations are muscle and soft tissue hematomas, often extensive, which contrasts with congenital hemophilia A (HA) [1]. Bleeding complications at diagnosis tend to recur as long as the autoantibodies are present. The rarity, lack of awareness, and nonspecific symptomatology frequently delay diagnosis and effective management [2].

Hemostatic treatment relies on bypassing agents not recognized by the anti-FVIII antibodies. These include recombinant activated FVII (rFVIIa), activated prothrombin complex concentrate, or porcine rFVIII (rpFVIII). These hemostatic agents, administered intravenously, have a short half-life, justifying repeated administrations. Their hemostatic efficacy is difficult to assess by routine laboratory tests. They are not without thrombotic risk [3]. Consequently, they are administered only in cases of severe hemorrhagic complication or during even minor invasive procedure, either on demand or for brief prophylaxis periods. Their pharmacokinetic properties do not allow for prolonged prophylaxis until the eradication of autoantibodies [4].

These multiple limitations of currently used hemostatic agents necessitate the use of immunosuppressive treatment (IST). Its goal is to eradicate the autoantibodies responsible for the inhibition of endogenous FVIII. This treatment is generally administered without delay upon diagnosis in all patients. It involves common immunosuppressive agents (corticosteroids, cyclophosphamide, and rituximab) alone, in combination, or sequentially according to various modalities [3,5]. The eradication of the inhibitor takes time and is not guaranteed, with some patients being refractory and exposed for a prolonged period to spontaneous or provoked hemorrhagic complications, possibly fatal [6]. These treatments are not without the risk of infectious and metabolic complications. These complications are frequent among weakened older patients and add to the hemorrhagic complications [7]. Considering the high mortality from bleeding or complications of IST, the current dual therapy for AHA is not optimal.

The recent development and validation of a new bypassing agent, emicizumab, a bispecific antibody mimicking the action of FVIII, has revolutionized the management of HA [8]. Inspired by the success of this new agent, especially in congenital HA with inhibitors, several groups have evaluated emicizumab among persons with AHA [9]. Not surprisingly, its efficacy was confirmed, including in a recent scoping review where participants benefited from emicizumab prophylaxis [10]. While the hemostatic benefit of using emicizumab in AHA is almost beyond doubt, it is its earliest possible initiation that seems to be emerging as the therapeutic modality of choice [11].

Emicizumab’s ability to bypass FVIII with consistent hemostatic efficacy through infrequent subcutaneous injections eliminates the need for rapidly initiated IST. For persons with AHA, emicizumab allows for the postponement of IST, just as it allows for delaying immune tolerance induction in persons with congenital HA and inhibitors [12]. By analogy, if systematic prophylaxis is formally indicated in persons with congenital HA with inhibitors, what reason could justify not to initiate prophylaxis in persons with AHA, especially older with multiple comorbidities, who are at an increased risk of predictable (recent surgery or invasive procedure, peptic ulcer, etc.) or unpredictable hemorrhagic complications that can be severe and potentially life-threatening/limb-threatening.

Considering these points, it seems reasonable to initiate emicizumab treatment early for any person diagnosed with AHA. Due to its pharmacokinetic and pharmacodynamic characteristics, emicizumab is not indicated for treating acute bleeds. In one of the first reported case series, after the first dose of emicizumab, the activated partial thromboplastin time normalized in 1 to 3 days, FVIII (human reagents) exceeded 10% after 11 (7.5-12) days. Hemostatic efficacy was obtained, and bypassing therapy stopped after 1.5 (0.8-4) days/IQR [9]. More recently, a phase 3 multicentric study (AGEHA protocol) has yielded encouraging outcomes through the implementation of an adjusted regimen, including a loading dose of emicizumab, distributed across 2 days (6 mg/kg on day 1 and 3 mg/kg on day 2), and 1.5 mg/kg once weekly from day 8 onwards [13]. The aim was to truncate the timeline to achieve maximum efficacy, guided by dose calculations informed by pharmacokinetic simulations and suggested steady-state emicizumab concentration after 1 week. This dosing protocol, validated through an open-label, single-arm, phase 2 clinical trial involving 47 persons with AHA in Germany, has confirmed efficacy and allowed deferral of IST for the first 12 weeks after diagnosis [14].

Based on available data, promptly initiating emicizumab for hemostatic prophylaxis in persons with AHA, regardless of the presence of hemorrhagic symptoms, offers multiple benefits (Figure):

1. Starting emicizumab immediately after diagnosing AHA can reduce the incidence or recurrence of bleeding and may improve control of active bleeding identified at diagnosis.
2. The hemostatic effect of emicizumab may reduce or eliminate the need for traditional adjuvant bypassing agents, which can be combined safely with emicizumab, such as rFVIIa and rpFVIII.
3. Emicizumab’s ability to decrease bleeding risk could delay or reduce IST aimed at autoantibody eradication. Tailored IST could lead to fewer adverse events, making it an especially beneficial approach for patients with multiple comorbidities.
4. Postponing IST should facilitate systematic identification and management of comorbidities that might impact the tolerance to IST. It could enhance the performance of the etiologic work-up of AHA (eg, minimizing interference by corticosteroids and enabling safer invasive procedures) and avoid potential interactions between IST and the treatment of the underlying disease.
5. Utilizing emicizumab should shorten hospital stays and lessen physical deconditioning, frequent among old persons with AHA, and potentially mitigated further by rehabilitation programs feasible with emicizumab.

**Figure:**
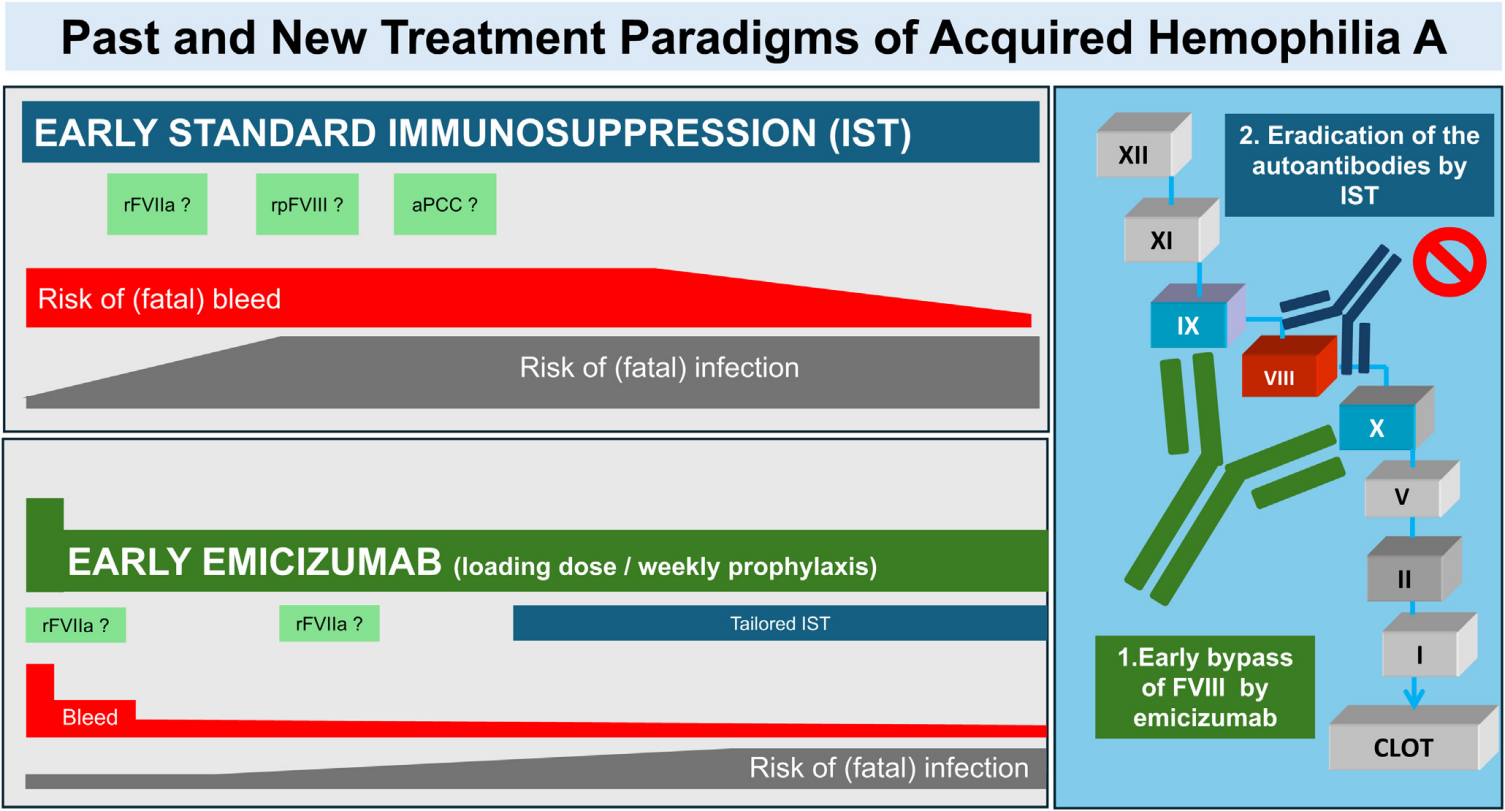
The aim of acquired hemophilia A treatment is 2-fold: to stop the bleeding and to eradicate the inhibitor. Current treatment includes systematic immunosuppressive treatment (IST) initiated early to eradicate the inhibitor and intravenous bypassing agents given on demand or for short prophylaxis. This approach is associated with a persistently high risk of possibly fatal bleeding and infectious complications. Rapid emicizumab loading initiated as first-line therapy at time of diagnosis and continued prophylactically appears as a safe and effective hemostatic approach, allowing to postpone and tailor IST to each patient’s profile and condition. APCC, activated prothrombin complex concentrate; rFVIIa, recombinant activated factor VII; rpFVIII, recombinant porcine factor VIII.

While there are compelling reasons to support the early adoption of emicizumab in persons with AHA, it is important to recognize that this therapeutic approach has its limitations, and several important considerations must be considered:

1. Emicizumab primarily serves as a preventive, long-term measure rather than a treatment for acute bleeding episodes. Nonetheless, administering higher-loading doses has been found to provide rapid hemostatic effectiveness.
2. Thrombin generation studies conducted *in vitro* have shown that emicizumab mimics a FVIII activity level of 10 to 15 IU/dL [8,15]. As a result, emicizumab improves the clinical bleeding phenotype from severe to mild, but adjuvant therapy with rFVIIa (or rpFVIII) is required for bleeding manifestations or invasive procedures with a significant risk of bleeding [4]. As a result, unlike persons with congenital HA who learn to cope with a bleeding risk from early childhood, persons with AHA require education to recognize and prevent bleeds.
3. The utilization of emicizumab necessitates specialized laboratory expertise due to its potential to interfere with standard clotting assays. This interference necessitates the employment of bovine-derived chromogenic reagents for the accurate assessment of FVIII activity and inhibitor titers. This limitation should consider that diagnosis of AHA (FVIII assay and inhibitor titration) and monitoring of its current treatment are not routinely available in many institutions [16].
4. It is noteworthy that, although rare, the development of alloantibodies against emicizumab has been documented in persons with HA and could likely occur in those with AHA. This should be monitored in the future as autoimmune diseases may increase the likelihood of developing antidrug antibodies [17].
5. The administration of emicizumab is currently not advised for pregnant and breastfeeding women.
6. The potential thrombotic risk associated with the concurrent use of bypassing agents requires cautious consideration. Although the HAVEN 1 study suggests a favorable safety profile when using rVIIa alongside emicizumab in persons with congenital HA, prior treatment with activated prothrombin complex concentrate (which should be ruled out) typically contraindicates the use of emicizumab in most persons with AHA, or it should be administered at the lowest feasible dose (<50 IU/kg) [8]. Additionally, the thrombotic risk during FVIII normalization with IST while on emicizumab is yet to be fully understood, with a wide FVIII discontinuation threshold range of 10 to 86 IU/dL [4]. Insights from the recently published prospective clinical trials in Europe [14] and the ongoing study in the United States (NCT05345197) are expected to provide guidance on emicizumab discontinuation thresholds. Like in persons with congenital HA, an additive or synergistic effect of emicizumab with endogenous FVIII is not anticipated [10]. In this context, the thrombotic risk should be considered for every patient before starting treatment with emicizumab; increased vigilance is warranted, particularly when initiating treatment in elderly patients with multiple comorbidities.
7. Except for Japan, emicizumab has not received official approval for this specific indication. Nevertheless, in certain countries, reimbursement may be attainable through individual patient case assessments by insurance providers.
8. Not all hospitals have access to emicizumab that could be rapidly provided to persons with AHA. Furthermore, many physicians outside of the hemophilia specialty are not yet acquainted with this treatment approach. Initiatives to increase awareness and foster good communication and collaborative care between hemophilia specialist centers and other institutions are essential.
9. The pharmacoeconomic impact of early emicizumab administration should be assessed, with consideration given to the reduced need for other bypassing agents, the decrease in hemorrhagic and infectious complications, and the shortened hospital stays.
10. The dosing protocols for emicizumab in this context, as well as the appropriate IST regimens when combined with emicizumab, require clearer definition.

The advent of emicizumab has significantly transformed the treatment landscape for congenital HA, exemplified by its role as a primary therapy in very young, previously untreated patients with severe disease, as evidenced by the recent HAVEN 7 clinical trial [18]. This innovation hints at a similar transformative potential for the management of AHA. Recent studies have underscored emicizumab’s exceptional efficacy in preventing breakthrough bleeding in persons with AHA. The prompt initiation of emicizumab as a first-line treatment introduces a novel strategy for managing AHA, providing patients with fast, consistent, and effective hemostatic control. This approach also allows for the deferral of IST, which can be tailored and potentially less harmful, marking a significant step forward in treating this condition. Further studies are warranted to establish the optimal IST regimen, with particular attention on potential breakthrough bleeds.
